# Investigation of Possible Role of the PAR-2 Receptor in Intestinal Inflammation

**DOI:** 10.4103/0975-1483.62214

**Published:** 2010

**Authors:** MB Patel, MA Patel, GB Shah

**Affiliations:** 1 *Department of Pharmacology, Shree Sarvajanik Pharmacy College, Mahesana, India*; 2 *C.K.Pithawalla Institute of Pharmaceutical Science and Research, Surat, India*; 3 *K.B. Institute of Pharmaceutical Education and Research, GH-6, Sector 23, Gandhinagar, Gujarat, India*

**Keywords:** Trypsin, PAR-2 receptor, inflammatory bowel disease

## Abstract

The present study was undertaken to study the role of PAR-2 receptor activation in pathophysiology of intestinal inflammation. Inflammatory bowel disease was induced in Wistar albino rats by intrarectal administration of 2, 4, 6 trinitrobenzenesulfonic acid (TNBS, 0.25 ml 120 mg/ml in 50% ethanol intrarectally, on 1^st^ day only). Trypsin (500 μg/kg, 1 mg/kg, 5 mg/kg, intrarectal) was given from the same day up to 20 days. Various physical parameters including body weight, food and water intake were measured on 1^st^ and 20^th^ days. At end of the experiment, colon weight and various histopathological indexes were assessed. The colon homogenate malondialdehyde (MDA), myeloperoxidase (MPO), and superoxide dismutase (SOD) and % mast cell protection in mesentery were also measured. Trypsin at higher dose (5 mg/kg) showed the higher level of oxidative enzymes and lower level of protective enzymes as compared to the animals treated with only TNBS. Trypsin treatment produced significantly more mast cell degranulation. Finally in the histopathology, there was increased in severity of the disease in trypsin-treated animals. The role of PAR-2 (protease activated receptor-2) receptor in gut is pro-inflammatory and thus appears as a new potential therapeutic target for inflammatory bowel disease treatments.

## INTRODUCTION

Inflammatory bowel disease encompasses a number of chronic, relapsing inflammatory disorders involving the gastrointestinal tract.[1] The two primary types of inflammatory bowel disease are Crohn’s disease and ulcerative colitis. In inflammatory bowel diseases, the intestine (bowel) becomes inflamed, often causing recurring abdominal cramps and diarrhea. Although the exact cause of ulcerative colitis remains undetermined, the condition appears to be related to a combination of genetic and environmental factors. Among the pathological findings associated with inflammatory bowel disease are increases in certain inflammatory mediators, signs of oxidative stress, a deranged colonic milieu, abnormal glycosaminoglycan (GAG) content of the mucosa, decreased oxidation of short chain fatty acids (SCFAs), increased intestinal permeability, increased sulfide production, and decreased methylation. While not one factor has been identified as the initial trigger for inflammatory bowel disease.[2] Proteinase-activated receptors (PARs) have the common property of being activated by the proteolytic cleavage of their extracellular N-terminal domain. The new NH_2_^−^ terminus acts as a ‘tethered ligand’ binding and activating the receptor itself. Four members of this family have been cloned, three of which are activated by thrombin (PAR-1, PAR-3 and PAR-4) while the fourth (PAR-2) is activated by trypsin or mast cell tryptase.[3] In physiological or pathophysiological conditions, the gastrointestinal tract is exposed more than other tissues to proteinases (digestive enzymes, proteinases from pathogens or proteinases from inflammatory cells) that can activate PARs. Since PARs are highly expressed throughout the gastrointestinal tract,[4] the study of the role of PARs in these tissues appears to be particularly important. In inflammatory or allergic conditions, the proteinases that constitute the major agonists for PARs (thrombin, trypsin, and mast cell tryptase) are usually released.[5] Protease-activated receptor-2-activating peptides induce leukocyte rolling, adhesion, and extravasations.[6] PAR receptor agonist also involve in release of mediators from mast cell[7] which have a role in development of inflammation. The activation of PARs by these proteinases might contribute to the gastrointestinal inflammation.[8] Particularly PAR-2 receptor is involved in development of inflammation.[9] The role of PAR-2 during intestinal inflammation is still unclear due to the fact that PAR-2 activating peptide had both pro- and anti-inflammatory properties. PAR-2 is strongly expressed in the small intestine, colon, liver, pancreas and more weakly detected in the stomach. PAR-2 is also highly expressed by human colon adenocarcinoma cells.[10] This study was carried out to investigate the role of PAR-2 activation in the process of inflammation in the gut.

## MATERIALS AND METHODS

### Animals

Adult albino (Wistar strain) rats of either sex weighing between 200 and 250 g housed in standard conditions of temperature (22 ± 2°C), relative humidity (55 ± 5%), and light (12 h light/dark cycles) were used. They have been fed with standard pellet diet and water *ad libitum*. Animal were approved by the Institutional Animal Ethics Committee (IAEC) according to the regulation of Committee for the Purpose of Control and Supervision of Experiments on Animals (CPCSEA). Throughout the experiments, animals were handled according to the suggested ethical guideline for the care of laboratory animals.

### Experimental protocol[11]

**Figure 1:**
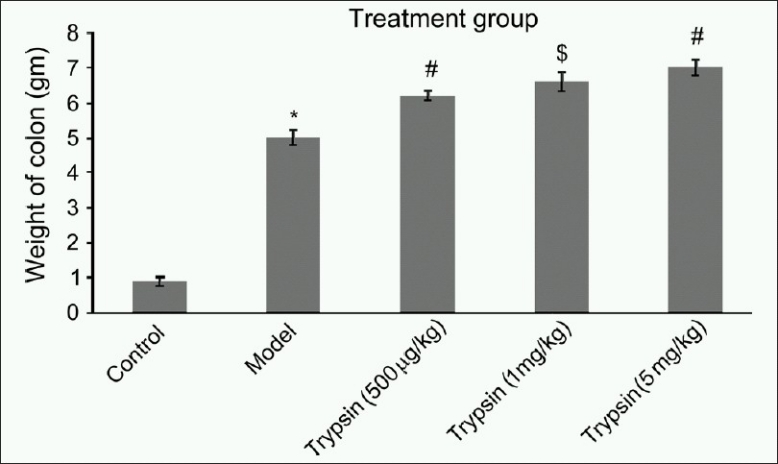
Effect of trypsin on the weight of the colon in TNBS-induced inflammatory bowel disease in rats. (Control is only saline treated, model group is only TNBS treated.); *When compared to control *P* < 0.001; ^#^When compared to model *P* < 0.001; ^$^When compared to model *P* < 0.01. One-way ANOVA (n = 6)

- Control: Saline treated;
- Model: TNBS (2, 4, 6-trinitrobenzenesulfonic acid, 0.25 ml, 120 mg/ml in 50% ethanol, intrarectally) on 1^st^ day only;
- Group 1: TNBS (0.25 ml, 120 mg/ml in 50% ethanol, intrarectally), on 1^st^ day only + Trypsin (500 μg/kg, intrarectal) treatment continued till 20^th^ day
- Group 2: TNBS (0.25 ml, 120 mg/ml in 50% ethanol, intrarectally) on 1^st^ day only + Trypsin (1 mg/kg, intrarectal) treatment continued till 20^th^ day
- Group 3: TNBS (0.25 ml, 120 mg/ml in 50% ethanol, intrarectally) on 1^st^ day only + Trypsin (5 mg/kg, intrarectal) treatment continued till 20^th^ day;

TNBS was delivered by a Teflon cannula (outside diameter 1.2 mm, inserted 8 cm) through the anus of each rat. Ethanol evokes an acute inflammatory response which resolves spontaneously after 1 week. Therefore, we preferred to include a saline-treated group as a negative control instead of an ethanol-treated group. Various physical parameters such as body weight, food intake, and water intake were measured on 1^st^ and 20^th^ days. On 20^th^ day, animals were sacrificed by cervical dislocation and dissected open to remove GIT (from stomach to anus). GIT was flushed gently with saline and cut open. Weight of the colon taken was measured and then the colon mucosa damage index (CMDI) [Figure 1] and the histopathological score i.e. disease activity index (DAI) were evaluated. Colon samples were taken for determinations of myeloperoxidase (MPO). Percentage protection of the mast cell degranulation in the mesentery of intestine of the rat was also measured.

### Assessment of the colon mucosa damage index

The colon segment taken 10 cm proximal to anus of the sacrificed rats was excised longitudinally, was rinsed with saline buffer and fixed on a wax block. Each colon was observed and evaluated by two independent observers. Macroscopic scoring was done evaluated according to the formula of CMDI reported by Wei *et al*., 2003.[12] Briefly describe as follows:[1] 0-normal mucosa, 1-mild hyperemia, no erosion or ulcer on the mucosal surface,[2] 2-moderate hyperemia, erosion appearing on the mucosal surface,[3] 3-severe hyperemia, necrosis and ulcer on the mucosal surface with the major ulcerative area extending <40%,[4] 4-severe hyperemia, necrosis and ulcer on the mucosal surface with the major ulcerative area extending >40%.

### Assessment of disease activity index

**Figure 2:**
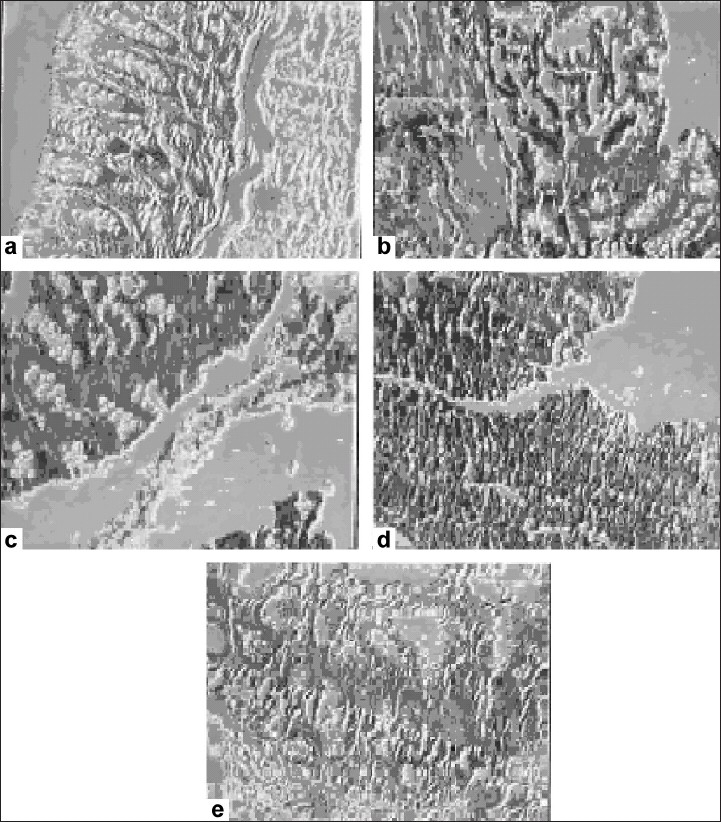
Tissue histopathology. Control group: Shows normal mucosa (a), Model group: Shows severe hyperplasia, ulcer appearing on the mucosal surface with the major ulcerative area extending. 40% (b), Group 1: Shows severe hyperplasia, ulcer appearing on the mucosal surface with the major ulcerative area extending. 50% (c), Group 2: Shows severe hyperplasia and ulcer appearing on the mucosal surface with the major ulcerative area extending. 55% (d), Group 3: Shows severe hyperplasia, ulcer appearing on the mucosal surface with the major ulcerative area extending. 60% (e)

The colon tissue samples taken for histology were fixed overnight in 4% neutral buffered formalin, processed, sectioned (4 μm thick), and stained with hematoxylin and eosin [Figure 2]. Each colon sample was observed and evaluated by two independent observers. To assess the histopathological score was assessed according to the modified model of the system reference given by Wei *et al*., 2003, which are as follows: (1) The infiltration of acute inflammatory cells: 0-no, 1-mild increasing, 2-severe increasing; (2) The infiltration of chronic inflammatory cells: 0-no, 1-mild increasing, 2-severe increasing; (3) The deposition of fibrotin protein: 0-negative, 1-positive; (4) the submucosa edema: 0-no, 1-patchy-like, 2-fusion-like; (5) the epithelium necrosis: 0-no, 1-limiting, 2-widening; (6) the epithelium ulcer: 0-negative, 1-positive.

### Determination of the myeloperoxidase in the colon

The colon sample was homogenized (50 g/l) in 50 mmol/l ice-cold potassium phosphate buffer (pH 6.0) containing 0.5% of hexadecyltrimethylammonium bromide. The homogenate was first frozen and thawed thrice for three times, and then centrifuged at 4000 rpm for 20 min at 4°C for the measurement of myelopeoxidase (MPO) activity. MPO, a marker of neutrophil migration was estimated by measuring H_2_O_2_^−^ dependent oxidation of O dianisidine.[13]

### % Mesenteric mast cell protection

% Mast cell protection is suggested by a decrease in the degranulation of the mast cell. Mesentery of intestine from obtained the animals was removed and placed in the Ringer Locke solution (NaCl 0.9%, KCI 0.042%, CaCl_2_ 0.024%, NaHCO_3_ 0.015%, and dextrose 0.1%). Then it was stained and fixed with the 4% formaldehyde containing 0.1% toluidine blue. % Mast cell protection was evaluated microscopically at ×40 magnification.[14]

### Statistical analysis

Data obtained from the animal experiments were expressed as the mean ± SEM of six observations. The statistical difference was evaluated by one-way ANOVA. Differences were accepted as statistically significant when *P* < 0.05.

## RESULTS

On 1^st^ and 21^st^ days, changes in the physical parameters including body weight, food intake, and water intake were measured [Table 1]. When compared with the control group, significant reductions in all these parameters were observed with the model group. However, in the animals treated with trypsin, still high reduction in the body weight, food intake and water intake was found compared to the model group.

**Table 1:** Effect of trypsin on water intake, food intake, and body weight in TNBS-induced inflammatory bowel disease in rats

Groups	Reduction in body weight (g)	Reduction in food intake (g/group)	Reduction in water intake (ml/group)
Saline treated	7 ± 1	5 ± 0.8	5 ± 1.8
Only TNBS treated	50 ± 2.5*	50 ± 4*	45 ± 1.2*
Trypsin (500 μg/kg)	59 ± 2#	57 ± 3.9#	54 ± 2#
Trypsin (1 mg/kg)	61 ± 2.9#	60 ± 5.2#	58 ± 1.1#
Trypsin (5 mg/kg)	72 ± 3.5#	66 ± 4.4#	63 ± 2.1#

Each value presented as mean ± SEM (n = 6) (one-way ANOVA)

* Compared with control group, *P* < 0.001;

# Compared with model group, *P* < 0.001

Animals treated with TNBS showed high colon weight compared to saline-treated animals. Animals treated with trypsin showed high colon weight compared to only TNBS treated. That means trypsin treatment increase severity of inflammation.

The main parameters used for evaluating the degree of colonic inflammation in IBD were CMDI, DAI. In this study, significant increases in CMDI and DAI were found, when compared with that of only TNBS-treated animals [Table 2].

**Table 2:** Effects of trypsin on CMDI, DAI, and MPO activity in the colon tissue of TNBS-induced inflammatory bowel disease in rats

Groups	CMDI	DAI
Saline treated	0.0 ± 0.0	0.7 ± 0.11
Only TNBS treated	6.1 ± 0.31*	8.3 ± 0.2*
Trypsin (500 μg/kg)	7 ± 0.21#	9.2 ± 0.10#
Trypsin (1 mg/kg)	7.8 ± 0.13#	9.9 ± 0.13#
Trypsin (5 mg/kg)	8.5 ± 0.18#	11.5 ± 0.11#

(CMDI: Colonic mucosal damage index, DAI: Disease activity index); Each value presented as mean ± SEM (n = 6) (one-way ANOVA)

* when compared to control group *P* < 0.001

# when compared to model group *P* < 0.001.

Animals treated with typsin showed high MPO activity compared only TNBS treated animals [Table 3]. Also there is high mast cell degranulation with trypsin treatment compare to TNBS alone [Table 3].

**Table 3:** Effect of trypsin on myelopeoxidase and mast cell degranulation in TNBS-induce inflammatory bowel disease in rats

Groups	MPO (unit/mg of protein)	Mast cell degranulation (% protection)
Saline treated	22 ±0.9	80 ±1.5
Only TNBS treated	68 ±2.5*	15 ±0.5*
Trypsin (500 μg/kg)	79 ±1.2#	11 ±1.0#
Trypsin (1 mg/kg)	90 ±15#	9 ±0.8#
Trypsin (5 mg/kg)	99 ±1.8#	7 ±0.9#

Each value presented as mean ±SEM (n = 6) (one way ANOVA)

* Compared with control group *P* < 0.001

# Compared with model group *P* < 0.001

## DISCUSSION

TNBS is a hapten compound, and when it is bound with a substance of high molecular tissue proteins, it will turn into an antigen. It has shown that it can elicit immunologic responses, induce generation of inflammatory bowel disease (IBD).[15,16] This model shares many of the histopathological and clinical features of human IBD and is useful for the study of the etiopathogenesis of chronic colon inflammation as well as providing an inexpensive model suitable for assessing therapeutic agents.

In IBD, body weight, food intake, and water intake are the important indicators of the severity of this disease. As there is a severe inflammation in the colon, the tolerability to the food and water decreases, and therefore body weight also decreases.[17] In our study we found that the treatment with trypsin decreased food and water intake. Weight loss is also increased.

In IBD, weight of colon is increased due to severe inflammation and edema.[18] Animal treated with TNBS showed high colon weight compare to normal animals. Treatment with trypsin increased colon weight of animals compared to animals treated only with TNBS. That shows that typsin increase inflammation and edema.

In our study, we induced IBD by intrarectal administration of TNBS in animals. The severity of colonic inflammation in developed disease was evaluated by measuring main parameters CMDI and DAI scores and MPO activity. In present study, we found that increased in progression of the disease pathogenesis following treatment with trypsin characterized by significantly increased in the score of CMDI and DAI compared to the model group which is also supported by the changes in the histopathology of the colon. MPO is an enzyme found in the neutrophils and can be used as a quantitative index of inflammation in colonic tissue. MPO activity may be regarded as an index of inflammation damage. The main pathological feature of IBD is transmural infiltration of polymorphonuclear neutrophils and MPO is released from these neutrophils.[19,20] Treatment with trypsin significantly increased the level of MPO compared to the model group which indicates that trypsin increases the infiltration of the inflammatory cells which are responsible for increasing the progression of the disease condition.

Mast cell degranulation causes mucus secretion, mucosal edema, increased gut permeability, and release of various inflammatory mediators that may be responsible for some of the signs and symptoms of inflammatory bowel disease.[21] In our study, a significant rise in the mast cell degranulation was observed in the TNBS-treated animals, while in trypsin-treated animals, mast cell degranulation was significantly higher. That is because trypsin increased mediator release from mast cell through PAR-2 receptor.[7] This observation clearly indicates that trypsin increase severity of the injury produced by inflammatory mediators released from the mast cell degranulation.

## CONCLUSION

From the results of the present study, it can be concluded that trypsin increases the severity of the IBD and this may be because of the action of trypsin on PAR-2 receptors present in colon. PAR-2 receptor may have important role in inflammation of gut. Further study using PAR-2 antagonists is required to carry out to establish role of PAR-2 in intestinal inflammation.
